# Why are pregnant women physically inactive? A qualitative study on the beliefs and perceptions about physical activity during pregnancy

**DOI:** 10.1590/0102-311XEN097323

**Published:** 2024-01-08

**Authors:** Helen Gonçalves, Ana Luiza Gonçalves Soares, Marlos Rodrigues Domingues, Andréa Damaso Bertoldi, Maiara Gonçalves dos Santos, Mariângela Freitas da Silveira, Carolina de Vargas Nunes Coll

**Affiliations:** 1 Universidade Federal de Pelotas, Pelotas, Brasil.; 2 MRC Integrative Epidemiology Unit, University of Bristol, Bristol, U.K.

**Keywords:** Physical Activity, Pregnancy, Communication Barriers, Qualitative Research, Atividade Física, Gravidez, Barreiras de Comunicação, Pesquisa Qualitativa, Actividad Física, Embarazo, Barreras de Comunicación, Investigación Cualitativa

## Abstract

This study aimed to describe the beliefs and perceptions of pregnant women and healthcare providers about physical activity during pregnancy. Using a qualitative approach, 30 pregnant women and the 14 healthcare providers caring for them were interviewed in the second trimester of pregnancy. We included women who maintained, decreased, or stopped physical activity since becoming pregnant. They were divided into low (≤ 8 years) and high schooling (> 8 years). Semi-structured, in-depth interviews were conducted and guided by three key questions: (1) When does physical activity during pregnancy start to be considered a wrong behavior?; (2) What are the main barriers (biological or others) to physical activity?; and (3) Do the actions of healthcare providers and people close to pregnant women reinforce barriers? Interviews were audio recorded, transcribed, and analyzed based on recurring themes. All women changed their physical activity behavior (decreased or stopped) when they discovered their pregnancy. Fear of miscarriage, contractions, bleeding, and of causing malformations in the baby were the most reported reasons for decreasing or stopping physical activity. Participants also lacked access to consistent information and healthcare providers’ support on the benefits of physical activity. Despite the current international recommendations to regular physical activity during pregnancy, uncertainty regarding its benefits remains. Interventions to promote physical activity during this period should include the training of healthcare providers so they can advise and discard ideas contrary to mother-child health benefits.

## Abstract (pt)

Este estudo objetivou descrever as crenças e percepções de gestantes e profissionais de saúde em relação a prática de atividade física durante a gestação. Usando uma abordagem qualitativa, 30 gestantes e 14 profissionais de saúde que atendiam essas mulheres foram entrevistados durante o segundo trimestre de gravidez. Foram incluídas mulheres que mantiveram, diminuíram ou pararam de praticar atividade física desde o início gravidez. Elas foram divididas em baixa (≤ 8 anos) e alta escolaridade (> 8 anos). Foram conduzidas entrevistas semiestruturadas e aprofundadas, orientadas por três perguntas-chave: (1) Quando a atividade física durante a gestação passa a ser considerada um comportamento errado? (2) Quais as principais barreiras (biológicas ou outras) para a prática de atividade física? (3) Estas barreiras são reforçadas pelas ações dos profissionais de saúde e das pessoas próximas à gestante? As entrevistas foram gravadas em áudio, transcritas e analisadas a partir de temas recorrentes. Todas as mulheres mudaram a prática de atividade física (diminuíram ou pararam) quando souberam da gravidez. O medo de aborto espontâneo, contrações e/ou sangramento e o medo de causar malformações no bebê foram os motivos mais relatados para diminuir ou parar a atividade física. Os participantes também não tiveram acesso a informações confiáveis e apoio dos profissionais de saúde sobre os benefícios da atividade física. Apesar das recomendações internacionais atuais para a prática regular de atividade física durante a gestação, a incerteza quanto aos seus benefícios continua sendo comum. As intervenções para promover a atividade física durante esse período devem incluir o treinamento de profissionais de saúde para que eles possam aconselhar e descartar ideias contrárias aos benefícios para a saúde materna e infantil.

## Abstract (es)

El objetivo de este estudio fue describir las creencias y percepciones de mujeres embarazadas y profesionales de la salud con respecto a la práctica de actividad física durante el embarazo. Utilizando un enfoque cualitativo, se entrevistaron 30 mujeres embarazadas y 14 profesionales de salud que cuidaban a estas mujeres durante el segundo trimestre del embarazo. Se incluyeron mujeres que mantuvieron, redujeron o dejaron de practicar actividad física desde el inicio del embarazo. Ellas se dividieron en baja (≤ 8 años) y alta escolaridad (> 8 años). Se realizaron entrevistas semiestructuradas y en profundidad, guiadas por tres preguntas clave: (1) ¿Cuándo se considera que la actividad física durante el embarazo es un comportamiento incorrecto? (2) ¿Cuáles son las barreras (biológicas u otras) principales para la práctica de actividad física? (3) ¿Estas barreras se refuerzan por las acciones de los profesionales de salud y de las personas cercanas a la mujer embarazada? Las entrevistas fueron grabadas en audio, transcritas y analizadas a partir de temas recurrentes. Todas las mujeres cambiaron la práctica de actividad física (redujeron o dejaron de practicarla) cuando descubrieron el embarazo. El miedo a sufrir un aborto espontáneo, contracciones y/o sangrado y el miedo a provocar malformaciones en el bebé fueron los motivos más relatados para reducir o dejar de practicar la actividad física. Los participantes también no han tenido acceso a informaciones confiables ni apoyo de los profesionales de salud sobre los beneficios de la actividad física. A pesar de las recomendaciones internacionales actuales para la práctica regular de actividad física durante el embarazo, la incertidumbre cuanto a sus beneficios sigue siendo común. Las intervenciones para promover la actividad física durante este periodo deben incluir la capacitación de profesionales de salud para que puedan aconsejar y descartar ideas contrarias a los beneficios para la salud materna e infantil.

## Introduction

Physical activity during pregnancy offers low risk and is considered beneficial for most women, although anatomical and physiological changes may entail adjusted exercise routines ^1^. Without abnormalities or contraindications, moderate-intensity physical activity for at least 20-30 minutes on most days should be recommended ^2^ ^,^ ^3^. Physicians’ counselling may influence physical activity in pregnancy ^4^, and healthy weight gain, maintenance of physical fitness, decreased risk of gestational diabetes, increase in psychological well-being, easier labor, and good mother-child relationship after birth configure some of the benefits that justify physical activity recommendation ^5^ ^,^ ^6^. However, few women are physically active during pregnancy ^5^ ^,^ ^7^ ^,^ ^8^. Most studies from high-income countries report the main reasons for pregnant women avoiding leisure-time physical activity ^7^: nausea, physical discomfort, leg cramps, increased body size ^5^ ^,^ ^9^ ^,^ ^10^, lack of knowledge about the benefits of physical activity, doubts regarding the safety of physical exercise during pregnancy, and incorrect information from relatives and even health personnel ^5^ ^,^ ^9^ ^,^ ^11^.

Sociocultural norms reinforce these misconceptions, such that the consequences and benefits of physical activity influence the way women in different contexts maintain or adopt new behaviors ^12^. A social expression, it highlights values of social and historical contexts and is subject to norms that influence knowledge and representations throughout life ^13^. The pregnant body also suffers this process of cultural construction. During pregnancy, more specific policies aimed at the mother-child health target the body. Pictures of postpartum women showing skinny bodies, massively featured in the media, can alter perceptions about physical activity during pregnancy, resulting in psychological barriers for those women who do not fit such stereotypes. Considering that the health field only partially discusses these views on health in pregnancy and physical activity engagement, it is believed that different socioeconomic contexts may influence the way women perceive physical activity and consider it risky, optional, or necessary. This study aims to discuss contemporary perceptions about physical activity during pregnancy from the perspective of pregnant women and prenatal care professionals, focusing on the barriers to being physically active.

## Methods

### Participants

This study is part of the 2015 Pelotas (Brazil) birth cohort study, a large population-based longitudinal study designed to assess many health aspects of all children born in the municipality of Pelotas, Rio Grande do Sul State, in that year, with the first follow-up starting in pregnancy ^14^. For this study, 30 pregnant women from the original cohort were invited to participate.

Women were recruited from the cohort database and considered eligible if in the second trimester of pregnancy and having answered positively to the following question: “Did you use to engage in physical exercises/sports before knowing you were pregnant?”, assessed in the first follow-up of the study, usually at the first or second trimester of pregnancy. To categorize physical activity status, three questions were used: (1) “Have you changed your exercise routine after finding out about your pregnancy?”, (2) “Do you think you are more or less active now compared to before your pregnancy?” and (3) “Have you decreased your activities or stopped exercising after knowing you were pregnant?”. Women who answered “no” to question 1 were allocated to the “remained active” group; those who answered “yes” to question 1 and “less” in question 2 were classified in the “decreased physical activity” group; and those who answered “less” in question 2 and reported having reduced physical activity in question 3, were classified in the “stopped physical activity” group. If their physical activity status changed during follow-up, women were allocated to the group according to their status. Women were also grouped according to education level: up to eight complete years (lower schooling) and above eight years (higher schooling). This is justified since more poorly educated women usually seek assistance in the public health system, whereas better educated women generally use private health services. We hypothesized that at public health facilities, to which less educated women usually go, physical activity counselling would be less frequent due to shorter consultations with physicians and because they are often followed by different physicians throughout their pregnancies. Hence, six groups were generated: (a) high schooling and remained active; (b) low schooling and remained active; (c) high schooling and decreased physical activity; (d) low schooling and decreased physical activity; (e) high schooling and stopped physical activity; (f) low schooling and stopped physical activity. This sampling also aimed to obtain greater heterogeneity among interviewees, considering individual characteristics available in the database that could influence behaviors (physical activity engagement and adherence to counseling for physical activity practice), such as pregestational body mass index (BMI), age, self-reported skin color, previous pregnancies, miscarriages, or abortions. Sample characteristics are shown in Table 1.

**Table 1:** Characteristics of pregnant women in this study according to physical activity status. Pelotas, Rio Grande do Sul State, Brazil, 2015-2016.

Characteristics	Remained active		Decreased physical activity		Stopped physical activity	
	Low schooling	High schooling	Low schooling	High schooling	Low schooling	High schooling
	n (%)	n (%)	n (%)	n (%)	n (%)	n (%)
Age (years)						
15-19	2 (40.0)	1 (20.0)	1 (20.0)	1 (20.0)	3 (60.0)	0 (0.0)
20-24	1 (20.0)	0 (0.0)	1 (20.0)	2 (40.0)	0 (0.0)	0 (0.0)
25-29	0 (0.0)	1 (20.0)	1 (20.0)	0 (0.0)	0 (0.0)	1 (20.0)
30-34	0 (0.0)	1 (20.0)	2 (40.0)	2 (40.0)	1 (20.0)	1 (20.0)
35+	2 (40.0)	2 (40.0)	0 (0.0)	0 (0.0)	1 (20.0)	3 (60.0)
Skin color *						
White	4 (40.0)	3 (30.0)	5 (100.0)	5 (100.0)	4 (40.0)	4 (40.0)
Others	1 (10.0)	1 (10.0)	0 (0.0)	0 (0.0)	1 (10.0)	1 (10.0)
Overweight (BMI ≥ 25.0)						
No	2 (20.0)	3 (30.0)	3 (30.0)	2 (20.0)	4 (40.0)	1 (10.0)
Yes	3 (30.0)	2 (20.0)	2 (20.0)	3 (30.0)	1 (10.0)	4 (40.0)
Previous pregnancy						
No	3 (30.0)	4 (40.0)	1 (10.0)	3 (30.0)	3 (30.0)	1 (10.0)
Yes	2 (20.0)	1 (10.0)	4 (40.0)	2 (20.0)	2 (20.0)	4 (40.0)
Previous miscarriage/abortion						
No	4 (40.0)	5 (100.0)	3 (30.0)	5 (100.0)	2 (20.0)	5 (100.0)
Yes	1 (10.0)	0 (0.0)	2 (20.0)	0 (0.0)	3 (30.0)	0 (0.0)

BIM: body mass index.

* 1 missing data.

To understand what healthcare providers recommended to pregnant women, the professionals providing antenatal care to our sample were identified. In total, ten physicians, six community health agents, and two nurses were mentioned. Health agents and nurses from the public health system were mentioned as those who advised the most about behaviors. Of the ten physicians mentioned, six were interviewed: three of whom worked in the public sector and three in private offices. The other physicians did not formally refuse to participate, however, they were never available after several attempts.

### Procedures

Data were collected using semi-structured, in-depth interviews based on three questions: (1) “When does physical activity during pregnancy start to be considered a wrong behavior?”; (2) “What are the main barriers (biological or others) to physical activity?”; and (3) “Do the actions of healthcare providers and people close to pregnant women reinforce barriers?”. All participants were asked the same questions while flexibility was maintained to explore participants’ responses in further details. Although the interviews included an introduction and other information about behavior during pregnancy, the questions about physical activity that guided the interviews are shown in Box 1.

**Box 1:** Questions about physical activity used to guide interviews with pregnant women and health personnel. Pelotas, Rio Grande do Sul State, Brazil, 2015-2016.

QUESTIONS TO PREGNANT WOMEN	1. Were you physically active before pregnancy? 2. After you got pregnant, have you exercised, or do you think about doing it? 3. People always say that pregnancy is a time to physically relax. What do you think of that? 4. Has the doctor discussed physical activity with you? Did he/she speak spontaneously or because you asked about it? 5. Why did they not bring up the subject? 6. What were the medical recommendations, to stop or change? When? 7. Did you understand the medical recommendations? 8. Does anyone encourage you to do or not do physical activity during pregnancy? What do they say? 9. How long do you think pregnant women can remain active? Why? What happens later? 10. What kind of physical activity could be done? And which could not? Why? 11. And what about your household chores, have you changed? 12. What are the fears of doing physical activity and what kind of harm could the baby suffer? 13. Are you ashamed of your appearance (going to the gym)? 14. Have you ever felt sick while exercising (during or before pregnancy)?
QUESTIONS TO HEALTH PERSONNEL	1. What are women’s main uncertainties and doubts? 2. What are the main structural issues of the facility to meet the demands of pregnant women? (i.e., no ultrasound equipment). 3. What is usually said/advised regarding diet and physical activity? 4. Do pregnant women already arrive with information on certain subjects, which they found on the internet or from family, friends, or neighbors? 5. What would be a contraindication to a woman exercising during pregnancy? 6. What kind of risk is expected? (Mention examples of cases from the interviews, reported facts). 7. Do you indicate any source of information about exercising to women?

Interviews were conducted from September 2014 to October 2015 by a female interviewer with a background in psychology and experience in qualitative research but no interests in the research topic. No author was involved in the conduction of interviews. All interviews were home-based, audio-taped, and carried out in privacy after an initial telephone contact. Up to three interviews were conducted with each woman until data saturation was obtained. The first interview took place with an average of 24 gestational weeks. The second and third interviews were conducted at the end of the third trimester of pregnancy or, in some cases, soon after delivery. In total, two pregnant women declined from participating after the first interview without explaining their reasons.

Healthcare providers were only contacted when interviews with pregnant women were nearing completion, especially because many women changed their prenatal clinic. Moreover, this strategy prevented healthcare providers from identifying participants. All professionals were interviewed at their workplace once. The interviews were based on a topic guide mainly addressing their knowledge on physical activity guidelines during pregnancy (restrictions and indications). The questions about physical activity that guided the interviews with health personnel are shown in Box 1. All interviews were recorded and then transcribed by a trained person. Then, the content of each interview was discussed by the research team (interviewer and two researchers) regarding when new questions were posed to each woman and what to ask in the next contact. The interviews with healthcare providers were also discussed with the research team without further interviews.

### Analysis

Data were analyzed according to thematic analysis. Transcripts were independently read and coded by one author and the person who conducted the interviews to generate initial codes and search for main themes. Coding was an inductive and data-driven process informed by the main research questions. The themes and sub-themes were reviewed and refined in face-to-face meetings until reaching a consensus. Some interviewees’ narratives regarding physical ativity perceptions and barriers were selected. Chosen examples of participant quotes are shown throughout the text to illustrate the themes of this research.

## Results

As planned, our final sample included 30 women equally divided into groups according to their schooling: 15 in each group (high and low schooling). Regarding skin color, women were mostly white (n = 25), and 50% were overweight and in their first pregnancy. These distributions were intentional as our sampling process aimed to obtain heterogeneity and similarity with the cohort population from which these women were sampled.

Overall, women changed their physical activity behavior during pregnancy. Although this study aimed to understand differences according to behavioral changes regarding physical activity, it also focused on understanding if women from distinct educational backgrounds behave differently as many behaviors are associated with socioeconomic characteristics.

We found that, regardless of education level, all women were exposed to misinformation during pregnancy and that those around unfortunately disseminate these beliefs, including health personnel. The following section describes these changes and participants’ reasons for them according to the groups this study allocated them.

### Women who remained physically active during pregnancy

Of the five low-schooling women who reported maintaining physical ativity, three stopped it during the first trimester of pregnancy due to physical discomfort (pelvic pain, low blood pressure, and pain after walking). Of the two who maintained physical activity, one was strongly encouraged to continue these activities as she was part of an exercise intervention ^15^. However, she stopped physical activity at eight months of gestation because she had lost weight instead of gaining muscle mass, as (she) expected. The other woman was hospitalized at seven months of gestation due to fainting and received medical advice to quit physical activity.

Of the six pregnant women with high schooling who maintained physical activity, two discontinued activities; one felt very tired at eight months (shortness of breath and pain) during walking, and another, at seven months, had cervical dislocation and received medical recommendation to rest. The remaining three women continued physical activity; two of whom always received guidance from physical education professionals and the other, in the second gestational trimester, replaced Pilates due to bleeding by light walking. 

### Women who decreased physical activity during pregnancy

Among the nine women who decreased physical activity (five with low schooling and four with high), two situations were more common: (1) stopping regular physical activity or (2) choosing light walking.

In the low-schooling group, three out of five women had problems during pregnancy: two had premature births and one was prescribed bedrest. One reported having decreased physical activity in early pregnancy - she quit lifting weights and the gym manager advised her to resume it only after delivery.

Of the four highly educated pregnant women in this group, only one interrupted physical activity (walking) at the end of the second trimester due to pain while exercising. The others, despite reducing physical actibity, remained active.

### Women who stopped physical activity during pregnancy

The nine women who stopped physical activity (predominantly low-intensity walking) did so after confirming their pregnancy. In total, seven gave the following reasons: having a “twisted uterus” or a “short cervix”, blood pressure alteration, toxoplasmosis, or loss of amniotic fluid. A poorly educated adolescent (15 years old) felt embarrassed to practice physical activity at school and received no support from her physical education teacher. Another participant in this group had a history of spontaneous abortions and preferred to remain inactive. None of the women who stopped physical activity reported having resumed physical activity during pregnancy regardless of education, even when, for example, their blood pressure had stabilized, except for one, who returned to commute by bicycle.

The women initially perceived physical symptoms as negative and harmful, characterizing them as the main reason for interrupting or decreasing physical activity regardless of education. During conversations these symptoms were suggested to be harmful to the baby’s health.

### When does physical activity in pregnancy start to be considered a wrong behavior?

For all women, confirming their pregnancy marked the moment to consider behavioral changes. Participants mentioned negative outcomes in pregnancies of women who lifted weights or made sudden movements.

At first, the greater chance of pregnancy “failing” in the first three months shows the frailty that imposes physical and emotional restrictions. Sickness, drowsiness, and dizziness, common at the beginning of this period, bring more changes and differentiated care. Jumping, carrying weights, weightlifting, contact sports, riding a bike/motorcycle, and running refer to some of the activities to be avoided, a socially reinforced idea.

As most women indicated, the approach of childbirth requires relieving the discomforts of the period and prioritize well-being, usually meaning inactivity. Only light walking (not always seen as formal physical activity), is usually adopted. Walking, in addition to being thought as less dangerous, would help to prepare the body for labor and reduce pain: “*I’ve been told to walk a lot to avoid pain and this kind of stuff to deliver* (...) *Then, when the time comes, I will not feel pain*”, participant C. (16 years, low schooling, decreased physical activity), said.

To compensate pregnancy-related efforts, women understand that physical activity can offer more dangers than benefits. Although socially empowered (able to generate a new life), there also exist the idea that they are physically fragile, reinforcing that physical activity should be delayed until after childbirth, or later. The participant P. (24 years, high schooling, decreased physical activity), said “*You don’t have the same agility you used to, you are carrying a 30-cm child inside your body, all this area spreads! Your uterus, everything dilates! It is not only you! I think that we must avoid doing some things, but not everything…*”. Later, she explained that women must understand that it is temporary and that they can go back to their routines later. However. many women say that taking care of the baby impairs selfcare practices, such as exercising.

All women chose light-to-moderate walking when recommended. They also perceived walking as part of family activities, leisure, or necessity. Moreover, walking helps to increase weight resistance and relieve babies’ internal pressure at the “birth canal”, helping to support body changes and facilitate delivery. Despite these advantages, if abdominal contractures, pelvic pain, or bleeding (credited to walking) occur during the exercise, exercise becomes a threat and should be discontinued. J. (17 years, low schooling, remained active) used to walk or commute by bicycle to work. As soon as she knew about her pregnancy, she abandoned her bicycle but continued walking. As she also needed to walk at work, increasing overall physical activity, she delivered after eight months. The participant S. (31 years, low schooling, decreased physical activity), and her physician understood that excessive activities accelerated labor: “*During work, I used to walk a lot. I believe that it was too much* (…). *She* [physician] *said it could be caused by walking, it dilated; I had contractions, and nearly two fingers of dilation! Interestingly, when hospitalized, her glucose blood levels were very high, and not even the physician mentioned that*”.

In summary, three recurrent ideas indicate that the physcical activity in pregnancy is a risky behavior. The first perceives the first months of pregnancy as fragile, considering physical activity an unusual movement that goes beyond recommendations and also demands an emotional effort. Thus, health gains can be postponed, avoiding damages such as potential miscarriages. The idea persists to the end of pregnancy, considering physical activity as inadequate due to body size and considering the chance that moving could harm the fetus.

We were unable to observe in the statements the fact that natural childbirth requires physical vigor and that the stronger the woman, the easier the process. Nobody mentioned that the postpartum period and caring for the baby is easier if the woman has physical strength and endurance.

### What are the main barriers (biological or others) to physical activity?

Physical activity behavior is determined by factors in multiple domains that are considered barriers and explain the decrease/interruption in physical activity engagement ^16^ ^,^ ^17^ ^,^ ^18^. The ecological models of health behavior point out that the physical environment influences physical activity and interventions would be more effective by considering these aspects ^19^ ^,^ ^20^. Thus, sociocultural, and environmental characteristics play a role in determining physical activity. In this study, psychological and biological barriers stood out and mostly explained why women reduced/interrupted physical activity during pregnancy.

Among the reported barriers, two occurred most often: (a) fear of miscarriage, contractions, and/or bleeding and (b) physical activity causing malformations.

### Fear of miscarriage, contractions, and/or bleeding

The notion that physical activity requires physical, muscular, and respiratory effort opposes the fact that the pregnant body should be entirely dedicated to the baby. This may partially explain studies indicating low levels of physical activity during pregnancy ^7^. All interviewees reported fear of contractions, bleeding, and miscarriage. Some excerpts from participants’ statements exemplify that: “*If I could choose, I think I would go to the gym but I can only consider exercising after the fourth month, not before that* (...). *It may hurt the baby: pressing the baby, like during the* [physical activity] *effort. Although they say you can exercise, you don’t know how your body will react, then I chose not to do it for precaution* (...) *for fear of a miscarriage and not hurt myself, I am pregnant after all! I should beware!* ‘− *And what about the Pilates classes, are you still going?’*. *No, because I had bleeding, my physiotherapist said I should better stop*. (M, 36 years, high schooling, stopped physical activity).

“*You push it hard, you get a contraction, and when you make a huge effort, everything dilates. Well, the doctor told me not to lift weights! Squatting a lot is bad for you because there’s the compression and it pushes the baby down* (...) *it may accelerate an earlier labor! He said: keep on doing it if you think it is good for you. If you are not comfortable, stop it, don’t force it* (...) *Then my belly was too heavy and I had contractions, then I quit, I was afraid. ‘*− *And now you’re only walking?’. I only walk*. (C., 28 years, low schooling, decreased physical activity).

“*I walked too much. I used to walk but not that much, right? Then I started walking a lot and by night my belly was hardening. I said: this is not normal! I went to the doctor, and he said I was having contractions! The baby was trying to come out*” (E., 32 years, low schooling, decreased physical activity).

For those with one or more of these conditions, the most frequent advice from healthcare providers was stop exercising and, if needed, stop household and occupational activities. A physician (public sector) reported a technical difficulty to justify their advice for physical activity interruption -having no good ultrasound scans to assess placental morphology. As a precaution, he prefers women to simply stop activities. Other healthcare providers also advised physical activity interruption because they felt insecure about the potential benefits of being active and feared ethical (lawsuits) issues and maternal frustrations.

Unlike walking, participants and healthcare providers perceived cycling as harmful during pregnancy. The reasons given by both include (1) the type of movement and location of the body as it enters in contact with bicycle forces, which press the uterus and may lead to abortions; (2) riding a bicycle requires constant force; (3) riding could rupture the placenta or cause bleeding; (4) the risk of falling or bumping or injuring the fetus/woman. Only women (rather than healthcare providers) mentioned that shaking during cycling could cause malformations. Box 2 shows some of these opinions.

**Box 2:** Barriers to physical activity reported by pregnant women regarding the potential harms of cycling.

BARRIERS	STATEMENTS
Type of movement and effort level	“*I think that’s too much effort! I believe it’s too much! Maybe if you take it slow* (...) *but I am not sure. The legs bend a lot. It demands strength*” (C., 31 years old, decreased physical activity, high schooling). “*Before leaving* [doctor’s appointment] *I asked: I miss riding my bike, could I get back on my bike?* [the reply was]) *- ‘If everything is ok with your pregnancy and nothing hurts, don’t change anything. Do not ride* (...)’ [and the doctor concluded] ‘*you’d better not risk it*”. *If it depended on me, I would keep riding*” (J., 26 years, stopped physical cativity, high schooling).
Miscarriage	“*I used to walk; I didn’t know I could not ride a bike. ‘* − *What did she [*neighbor] *say?’. She lost her first baby when riding a bike. My boyfriend’s cousin lost her baby riding a motorcycle. ‘- Why, did she crash?’. I don’t know, I didn’t ask her! My neighbor also said that because she was cycling constantly, I think she hit here* [pelvic area] *and lost the baby. ‘* − *And did you stop cycling?’. Yes* (...) *I was afraid* (...) *Sometimes I think it is not true, a myth, but I’d rather not risk!*” (J., 17 years, remained active, low schooling).
Bumping/Shaking	“*I am afraid of the impact from the bike saddle ‘- Could that harm you or the baby?’. Disturbing, moving, misplacing something here inside! When we are pregnant, we wonder, the bumps from riding, maybe hitting a pothole, a rock, I believe it is harmful, right? If you fall* (...) *I consider being pregnant and falling, there is no reason to take that risk. Also falling and getting injured, displacing the placenta, I don’t know, causing the uterus to rupture due to the impact...*” (M., 19 years, remained active, low schooling).
Malformations	“*After I was sure I was pregnant, I told her that it was not good to ride my bike in the beginning because the fetus is growing and could develop malformations. ‘* − *Did the doctor say that?’. Yes, and harm the baby. But I should wait a while, like until the sixth month, then I could ride again. But how can I ride now with such a big belly?* (...) *when the fetus is growing, he/she grows upside down,* [right?]*, or upright, I don’t know! It could hurt a body part, arms, legs. ‘* − *By pressing the baby?’. Yes, pressing and bending the limbs of the baby, causing a malformation* (...) *maybe a foot, a leg, an arm*”. (S., 31 years, decreased physical activiy, low schooling).

Given these possibilities and the reality of little counselling from professionals, physical activity engagement is absent in prenatal care. This also explains why, of the ten women who remained physically active, six replaced their usual routines by light activities such as walking.

### Do the actions of healthcare providers and people close to pregnant women reinforce barriers?

According to what women reported from counseling on exercising during prenatal care, two common attitudes describe medical procedures: (1) asking general questions about daily activities; (2) not asking about physical actvity/exercise. When questioned by women regarding physical activity during pregnancy, healthcare providers often advise light walking as the only safe type of physical activity.

According to the participants, among the healthcare providers, six neither asked nor advised them about physical activity and more than half advised light walking. The others only talked about it when questioned by physically active patients.

In the group that remained active and had low education, only one physician asked about physical activity and recommended that the woman continue as long as she was feeling well, three never mentioned exercise, and one took a more general approach to daily activities. To the latter, the pregnant woman replied that, among other things, she was going to the gym and was advised to interrupt it and carry on with walking.

Among the healthcare providers who assisted the most educated women, two specifically asked about physical activity: one warned that it configured an impact physical activity (gym activities) but without explaining potential negative consequences, and another stated no contraindications to physical activity in the absence of pain; two others also addressed the subject superficially, letting the patient discuss her behavior. After women reported it, one encouraged her to go to the gym with individualized supervision and another warned that “excessive” walking would harm the pregnancy. In this group, another professional did not ask about physical activity, but the patient asked about Pilates, and the physicians advised she could continue it monitored by a physical therapist.

Among those who decreased physical activity and belonged to the low-education group, a physician specifically asked about the practice of physical activity and, according to the woman, he was only interested because she was overweight; one did not ask; and three others asked superficially what they were doing and recommended walking. Among high schooling women, healthcare providers simply did not mention exercise but two recommended light walks when asked about the potential problems resulting from physical activity.

Among the low-schooling women who stopped physical activity, only one was advised by D., a physician: “*You know it’s good for you!*”. All other healthcare providers did not address physical activity. High schooling women who stopped exercising showed a similar behavior: four professionals did not ask and one recommended walking despite pain symptoms.

Some women understood that physicians never mentioned exercise due to their professional training and that older professionals fully ignore it. However, silence about physical activity, advising against, or reinforcing walking as the only safe activity reinforce myths and fears. A female physician (M.) said that she was also afraid during her pregnancy, including one of the fears mentioned by participants: “*During my second pregnancy, I used to ride a motorcycle. Then I had a bleeding, which I know is normal, but it happened soon after I rode my motorcycle*”.

In conversations with non-medical professionals from the public services, they mentioned that the population nearby is predominantly inactive. According to them, walking and cycling are mainly for commuting. They are not, therefore, considered properly physical activity serving health and/or aesthetic purposes. However, these professionals’ understanding reinforces that physical activity is not stimulated and encouraged in pregnancy. This partially explains why walking is so recommended, whereas cycling is considered a great danger - in addition to the exercise itself, it offers the risk of traffic injuries.

A healthcare provider, mentioned as an example of professional who advises regular physical activity practice, when asked if he knew of any pregnant women who had walked or cycled excessively and suffered any physical complication, said: “*Not that I am aware of! Also because we do not know what kind of problems would happen. We usually think about miscarriage. It is possible, too much time on the bike, bleeding, hitting a pothole, falling, an injury. I don’t know what could happen, but what if she falls and hits her belly?! It could result in an internal injury. I believe it could happen*” (B., a healthcare provider).

A physician pointed out another aspect that explains the lack of incentive regarding physical activity for young low-income pregnant women. In this group, pregnancy denotes a moment in the transition from the status of girls depending on their family to an independent woman. Therefore, the baby must be kept from any potential danger. Making no physical effort confirms the gradual transition of status, gaining respect and support from those who live close to the woman. Despite the physician discussing it, this idea may reflect a common behavior.

Also, based on the low-income population, J., a public sector physician, explains why professionals may choose to avoid counsel about behavior, he said: “*Yes, I believe we should instruct them! But here it’s very hard, they simply don’t exercise. ‘− Do you usually ask them, when they get here, if they do any kind of exercise?’. I do ask. ‘− Do you ask or only if they ask?’. I ask, but they have five children! There is no way they can exercise, they simply can’t!*”.

Another professional admits that he never encourages or educate women to be active during pregnancy as physical activity is seen as harmful for the population of public medical services: “*There is this general idea, this myth that it is harmful, increases miscarriage risks* (...). *These are myths. I usually say: if you are insecure about exercising, don’t do it!*”. J., a community health agent, was questioned if he felt confident to advise a pregnant woman about physical activity during pregnancy and he quickly replied: “*No*”. When the interviewer insisted, he said: “*Maybe walking. Perhaps it is something harmless.* ‘- *Have you heard that it is good to walk during pregnancy?’. Walking is natural to humans, right? It is not something we could tell people not to do. Unless it is a high-risk pregnancy, for example, but apart from that…*”. On the other hand, another health agent was unable to explain why she advised women to stop riding bikes at the beginning of pregnancy: “*I believe that in the first trimester you shouldn’t ride a bicycle. ‘− Why not in the first trimester?’. I don’t know why. But it feels right not to ride* (...) *we worry a lot more about motorcycles, right?* [speaking about traffic dangers, falls, etc.]” (F., health agent).

Thus, one may wonder if healthcare providers are more likely to advise physical activity to pregnant women with higher education. The prenatal care of eight high-schooling women occurred at public facilities and seven at private practices. We interviewed the only two private physicians who agreed to participate in this study. One of them, highly praised by two participants, highlights three exercises he feels safe to indicate: walking, water aerobics, or Pilates. To those going to the gym, C., a physician, says: “*I don’t know what kind of activity they do at the gym. Some lift weights that are too heavy for a pregnant woman... so if I don’t know what they do, I instruct them to give up weight training*”. Another physicians guaranteed he investigates physical activity and recommends it. Her patient, considered healthy, was instructed to avoid “impact” activities. The term impact generated doubt as it was not addressed in consultations, causing the interruption of this kind of training. The “impact” is usually misunderstood. “*‘− What is this impact thing?*’. *You know* (...) *I don’t know, I asked, I thought it meant pushing down, like pushing the baby out, don’t know, didn’t ask* (...) *I wondered, because vertical impact, maybe it could lead to preterm labor*” (C., 36 years, , high schooling, remained active). In these two examples, uncertainties and little conversation on the subject discouraged physical activity engagement.

## Discussion

Results showed that few women are physically active during pregnancy and that the lack of understanding about physical activity benefits is linked to conceptions about its social role and physiological changes. Unlike previous studies ^20^ ^,^ ^21^ ^,^ ^22^, behaviors in this study show that nearly all interviewed women and healthcare providers lack confidence to engage and recommend physical activity during pregnancy. Vague and conflicting information from healthcare providers and family members indicate disconnection between practice and clinical evidence and recurrent social misconceptions.

Despite its potential benefits, most physicians did not encourage patients to become active, only six advised it (if under supervision) or deemed it walking as more appropriate. Misconceptions about the benefits of physical activity in pregnancy by professionals involved in antenatal care is only part of the barriers and is not an exclusive finding of this study ^17^ ^,^ ^22^ ^,^ ^23^ ^,^ ^24^ ^,^ ^25^. In the general population, health-care counseling for physical activity has proven to be a cost-effective and successful method for increasing physical activity levels ^26^. Professionals share many common beliefs about pregnancy and the role of the pregnant woman, including that pregnancy is a moment of almost exclusive dedication to babies that must avoid testing the capacity of the body beyond gestation.

The main barriers this study observed are linked to the fear miscarriage, the risks of strength/movements felt in the womb, and the assumption of this responsibility when engaging in physical activity or advising it. The social pressure exerted on pregnant women about how they should behave was greater to preserve their bodies without worrying about changes in shape and weight since the extra demand from the exercise could be harmful. Participants also often mentioned their concern about responsibility for their actions. Possible negative consequences, in their imagination, ignore being a good “mother” or good caregiver/professional, evincing other health priorities, which somehow disagree with women who exercise for individual need and pleasure. Given the more negative than positive perceived possibilities, most women opted for either physical inactivity or light walking. Only the four highly educated women who maintained physical activity were more confident about the benefits of exercising but three of them adhered to lower intensity exercise routines. The responsibility allocated to women regarding how her actions may affect their babies’ health, family, and motherhood demands is significant for maintaining or incorporating new behaviors ^27^. Thus, they tended to adhere to more valued normative models of the female role in their context during pregnancy ^28^.

Participants excluded aesthetics from the reasons to remain physically active. This finding may stem from the embarassment of admitting that or the rejection of the “fitness compulsion” that imposes excessive discipline to women.

As for the limitations of this study, the design it employed shows potential biases that may originate from the moderator, who could somehow influence responses during conversations. Moreover, complex analyses challenges qualitative studies, but we have tried to make it as objective as possible within the group of authors. We do not think that sample size was an issue, especially because qualitative studies scarcely rely on sample size demands (for statistical reasons). Nonetheless, as the studied group stemmed from a population-based sample, we are unable to consider it as a convenience sample. On the other hand, we are aware that the extrapolation of our results requires caution.

According to guidelines, pregnancy should be the ideal time for behavior change and the adoption of healthy lifestyles by increased motivation and frequent medical supervision. However, changes entail health actions. This study observed a general misinformation about gestational physical activity in participants and physicians. In the population, misinformation seems to perpetuate itself as a belief among family and friends and, among professionals, in addition to the lack of at physical activity subject in academic training, they fear that indicating an activity may cause women to associate it with negative outcomes. Moreover, when women show other risk factors, it seems that if they are active in any way, the occurrence of any negative outcome will be linked to physical activity. The risk of miscarriage is higher in the first trimester but it is independent of women’s activity and no evidence supports the theory of increased fetal risk resulting from physical activity. It is noteworthy that health agents and nurses are the professionals that advise the most, and at the same time, curiously, physical education professionals have similar fears, contributing to the interruption of activities. The knowledge about physical activity benefits during pregnancy will enable women to choose what to do despite the myths and beliefs that surround this period of life.
